# Effect of music on hemodynamic fluctuations in women during induction of general anesthesia: A prospective randomized controlled multicenter trial

**DOI:** 10.1016/j.clinsp.2024.100462

**Published:** 2024-08-02

**Authors:** Jie Wang, Linghui Jiang, Wannan Chen, Zhiyao Wang, Changhong Miao, Jing Zhong, Wanxia Xiong

**Affiliations:** Department of Anesthesiology, Zhongshan Hospital, Fudan University, Shanghai, PRC

**Keywords:** Anesthesia, Hemodynamic, Preoperative, Anxiety

## Abstract

**Background:**

The authors aim to investigate the effect of music on hemodynamic fluctuations during induction of general anesthesia and reducing preoperative anxiety for women who underwent elective non-cardiac surgery.

**Methods:**

It is a multicenter, double-blind, randomized, parallel-group clinical trial. Patients were randomized 1:1 to either a Music Intervention group (MI) or a Control group (Control). The MI participants listened to their preferred music for more than 30 minutes in the waiting area. The State-Trait Anxiety Inventory (STAI) was used to measure anxiety levels in the groups, and hemodynamic parameters (Heart Rate [HR], Mean Arterial Pressure [MAP]) were continuously recorded before induction (T0), at loss of consciousness (T1), immediately before intubation (T2), and after intubation (T3). Intubation-related adverse events were also recorded. The primary outcome was the incidence of MAP changes more than 20 % above baseline during T0–T2.

**Results:**

A total of 164 patients were included in the final analyses. The incidence of MAP instability during T0–T2 was lower in the MI, and the 95 % Confidence Interval for the rate difference demonstrated the superiority of MI. HR instability was less frequent in MI participants both in T0–T2 and T2–T3. The overall incidence of preoperative anxiety was 53.7 % (88/164). After the music intervention, the mean score of STAI was significantly lower in the MI than in the Control, with a between-group difference of 8.01.

**Conclusions:**

Preoperative music intervention effectively prevented hemodynamic instability during anesthesia induction and significantly reduced preoperative anxiety in women undergoing elective non-cardiac surgery.

## Highlights

• Preoperative music intervention effectively prevented hemodynamic instability during anesthesia induction in women undergoing elective non-cardiac surgery.
• The overall preoperative anxiety incidence was 53.7 %, which was associated with a greater incidence of hemodynamic instability.
• Preoperative music intervention significantly reduced preoperative anxiety.

## Background

Approximately 25 %–80 % of patients experience anxiety before surgery, and the US National Institute of Mental Health reported that the prevalence of anxiety disorders is 60 % higher in women than in men.^1^ In addition, compared both with older women and with men, younger women are significantly more likely to experience preoperative anxiety.^2^ Perioperative stress and preoperative anxiety are associated with a number of negative clinical outcomes, including prolonged anesthesia induction, greater use of intravenous anesthetics during induction and maintenance, and more severe postoperative pain.^3^^,^^4^

Previous studies found that hypotension and hypertension were, respectively, prevalent in 26 % and 20 % of patients during anesthesia induction.^5^ The cardiovascular response to general anesthesia induction sometimes results in blood pressure fluctuations called “Alpine anesthesia”.^6^ Hypotension may lead to negative outcomes such as myocardial injury, stroke, acute kidney injury, and death.^7^ Conversely, laryngoscopy and tracheal intubation often cause hypertension which may cause myocardial infarction, heart failure, pulmonary edema, and cerebral and subarachnoid hemorrhage.^8^^,^^9^ Thus, preventing hemodynamic fluctuations minimizes the likelihood of certain harmful complications. Several drugs have been evaluated as possible stabilizers of the cardiovascular response, but none have produced hemodynamic stability during induction because of either inadequacy of effect or unwanted side effects.^10^

Music as a non-pharmacologic intervention can decrease preoperative anxiety, leading to safer and more cost-effective surgical care.^11^ Studies have shown that music can effectively reduce anxiety and perioperative anesthetic requirements.^12^ Music can also stimulate the dopamine pathway,^13^ the release of brain endorphins (with effects similar to those of morphine),^11^ and the unconscious autonomic response.^14^ To date, music has been researched in preoperative, intraoperative, and postoperative settings.^15^^,^^16^ However, despite the long history of the beneficial use of music, few studies have focused on hemodynamic changes, anesthetic requirements, and adverse events during induction of general anesthesia.

The authors therefore designed the present non-inferiority study to evaluate whether music can reduce preoperative anxiety and promote hemodynamic stability during induction of general anesthesia in women.

## Methods

### Design, setting, and participants

The authors used a two-group randomized controlled trial design. Patients scheduled for elective surgery under general anesthesia at Zhongshan Hospital, Shanghai, PRC, or Zhongshan Hospital Xiamen Branch, Xiamen, PRC, were eligible to participate. The study is registered in the Chinese Clinical Trial Registry (ChiCTR2000040254, http://www.chictr.org.cn/showprojen.aspx?proj=64383) (Trial period: Jan 1, 2021 ‒ Dec 31, 2022). This study was conducted in accordance with the CONSORT Statement rules.

The authors recruited women 18–65 years of age with no hearing impairment, a secondary school degree or higher, an American Society of Anesthesiologists Grade 1 or 2 physical status, and a need for general anesthesia for a planned elective surgery of 1–4 hours’ duration. Patients were excluded if they had a severe pre-existing health condition, including renal, hepatic, or cardiac dysfunction, or a pre-existing mental illness; if they were taking antipsychotics or sedative hypnotics or beta-blockers; or if they had a history of drug abuse; or suspected difficulty-airway for preoperative evaluation. Patients who could not read or understand the questionnaires used in the study were also excluded.

Once patients had given written informed consent for participation, they were randomized 1:1 to either the Music Intervention group (MI) or the Control group (Control) immediately upon entering the preoperative waiting area. An online computer program generated randomization numbers for the group assignments.

### Intervention

Once participants were settled in the preoperative waiting area, a researcher administered the State-Trait Anxiety Inventory (STAI) to determine each patient's anxiety level. The STAI has two sections, the State Anxiety Inventory (SAI) and the Trait Anxiety Inventory (TAI), each consisting of 20 questions that are scored from 1 to 4.^12^ The SAI evaluates the respondent's current state of anxiety (“How do you feel right now?”) by measuring feelings of apprehension, tension, nervousness, worry, and activation of the autonomic nervous system. The TAI evaluates “anxiety proneness”, including the respondent's calmness, confidence, and security in general.^17^ The STAI manual does not define cutoffs, but a cut point of 39–40 has been suggested to detect clinically significant symptoms.^18^ The authors set the cut point at 40, defining an SAI score of 40 or higher as a preoperative anxiety event. Noninvasive Blood Pressure (NIBP), Heart Rate (HR), and Respiratory Rate (RR) were monitored and recorded every 10 minutes in the waiting area.

Thereafter, patients in the MI were given noise-canceling earphones (Bose, Framingham, MA, USA) and an iPod Touch device (Apple, Cupertino, CA, USA) containing 2000 songs covering various music genres (e.g., blues, classical, country, gospel, jazz, rhythm, and Chinese popular or traditional music). During the preoperative period, until they entered the operating room, MI participants could choose the playlist of their preference. Their preoperative music listening time was controlled to exceed 30 minutes. Control participants received noise-cancelling earphones to diminish the influence of environmental noise, but they received no music. Before transfer to the operating room, MI participants stopped listening to music, and all participants were asked to complete the STAI questionnaire again.

### Anesthesia procedure

In the operating room, patients were monitored using electrocardiography, NIBP, pulse oximetry, and bispectral index (BIS system: Covidien, Boulder, CO, USA), which measures the depth of anesthesia. Values were recorded every minute.

To avoid the influence of factors that might cause hemodynamic fluctuations at intubation (e.g., duration of laryngoscopy, difficulty in airway management, etc.) potentially confounding the results, general anesthesia was induced by two anesthesiologists with more than 5-years of experience administering anesthesia. All researchers in the operating room were blinded to the participant's group assignment and STAI scores. Before induction of anesthesia, assistants recorded the average of three HR and NIBP measurements as the baseline (T0) values. General anesthesia induction was defined as the period from the start of administration to the successful completion of tracheal intubation.

The intravenous induction strategies used were these: Patients received propofol (AstraZeneca, London, UK) by target-controlled infusion (Marsh model) using a pump (RC2: Fresenius Kabi, Brézins, France). The target plasma concentration of propofol was initially set at 5.0 µg/mL-1. Loss Of Consciousness (LOC) was assessed repeatedly for 3–5s. Once a patient lost consciousness (T1), 2 µg/kg-1 fentanyl (Yichang Renfu Pharmaceutical Industry, Hubei, PRC) was administered. Patients were mechanically ventilated using a face mask with an oxygen flow rate of 8 L/min-1. Rocuronium 0.6 mg/kg-1 (Merck, Kenilworth, NJ, USA) was administered 2.5 min later. Endotracheal intubation was started 1.5 min after the rocuronium injection and completed within 2 min using a video laryngoscope (Zhejiang UE Medical Corp, Zhejiang, PRC). Hemodynamic parameters were recorded at T0, T1, immediately before intubation (T2), and immediately after intubation (T3); the lowest NIBP and HR in each period were also recorded. The time from induction start to LOC (TLoc) and to intubation completion (Tinduction), and occurrences of specific side effects during induction such as cough reaction, involuntary movement, myoclonus, laryngospasms, bronchospasms, and secretions, were also recorded.

During each operation, anesthesia depth was maintained with a target-controlled infusion of propofol to keep BIS values at 40–60. A bolus dose of fentanyl (50 µg) or hydromorphone (0.2 mg) was administered when necessary. NIBP, HR, and BIS were recorded every 5 min. Phenylephrine 40 µg or ephedrine 6 mg was administered to avoid a 30 % decrease in Mean Arterial Pressure (MAP) or systolic arterial pressure of less than 85 mmHg. Atropine was administered at a dose of 0.3 mg to maintain an HR of 50 bpm or more. A single injection of esmolol was used to forestall serious tachycardia or hypertension. Palonosetron hydrochloride 0.3 mg was used to prevent nausea and vomiting at the end of surgery. The tracheal tube was removed when patients fully regained consciousness and strength, after which they were admitted to the post-anesthesia care unit.

### Definition of hemodynamic instability

Because most hypertensive events occur during intubation, the authors divided the induction procedure into two periods: T0–T2 and T2–T3, with the lowest HR and MAP values during T0–T2 being recorded as LT0–T2. Hemodynamic Instability (HI) events were defined as changes in MAP or HR to more than 20 % above baseline in each period.

### Outcomes

The primary outcome was the incidence of MAP changes more than 20 % above baseline during T0–T2. The secondary outcomes were:

• Incidence of HR changes more than 20 % above baseline during T0–T2;
• Incidence of HI events during T2–T3;
• STAI scores in the waiting area;
• Physiologic variables (HR, MAP, RR) in the waiting area;
• Intubation-related adverse events (cough reaction, involuntary movement, myoclonus, laryngospasms, bronchospasms, secretions);
• TLOC and Tinduction;
• Effect-site Concentration (Ce) at LOC.

### Sample size

The sample size was calculated via PASS (version 2021). According to pretest analysis in Zhongshan Hospital Fudan University, the incidence of MAP instability during T0–T2 in the MI group is assumed to be 0.85 under the null hypothesis and 0.65 under the alternative hypothesis. Group sample sizes of 77 in both groups achieve 90 % power to detect a difference between the group proportions of -0.2. The proportion in the control group is 0.85. With an anticipated 5 % dropout rate, the sample size for each group is 82. The test statistic used is the one-sided Z-Test with un-pooled variance. The significance level of the test is 0.05.

### Statistical analysis

Data were analyzed using the *R* for Windows (version 3.5.3: The R Foundation for Statistical Computing, Vienna, Austria) and IBM SPSS Statistics (version 22.0: IBM, Armonk, NY, USA) software applications. Normality and homogeneity of variance were tested using the Shapiro-Wilk test and the Levene test. Categorical variables are reported as frequencies and percentages and were compared using the Fisher precision probability test or the Pearson Chi-Squared test. Continuous variables with a normal distribution are shown as means with standard deviation and were compared using the Student *t-*test with Bonferroni correction. Non-normally distributed continuous variables are presented as medians with interquartile range and were tested using the Mann-Whitney U-test. Analysis of Covariance (ANCOVA) was used to compare the SAI and TAI scores and the hemodynamic changes in the two groups. The superiority of MI was analyzed by comparing the 95 % Confidence Interval (95 % CI) of the rate difference; *p <* 0.05 was considered statistically significant for all analyses.

A logistic regression model was used to estimate the relative risks of preoperative anxiety and HI. The incidence was entered as a dependent variable Y in the logistic regression model and was coded as 0 for absent (did not occur) or 1 for present (occurred). The authors applied and compared two regression methods: Stepwise Logistic (SL) regression and Least Absolute Shrinkage and Selection Operator (LASSO) regression. For LASSO regression, the authors used the glmnet package in *R*. For stepwise regression, the authors used the Akaike information criterion to select the covariates. For LASSO regression, the authors used cross-validation to select λ. The authors calculated the area under the receiver operating characteristic curve to measure predictive performance for the fitted models.

## Results

### Baseline data

**Fig. 1:**
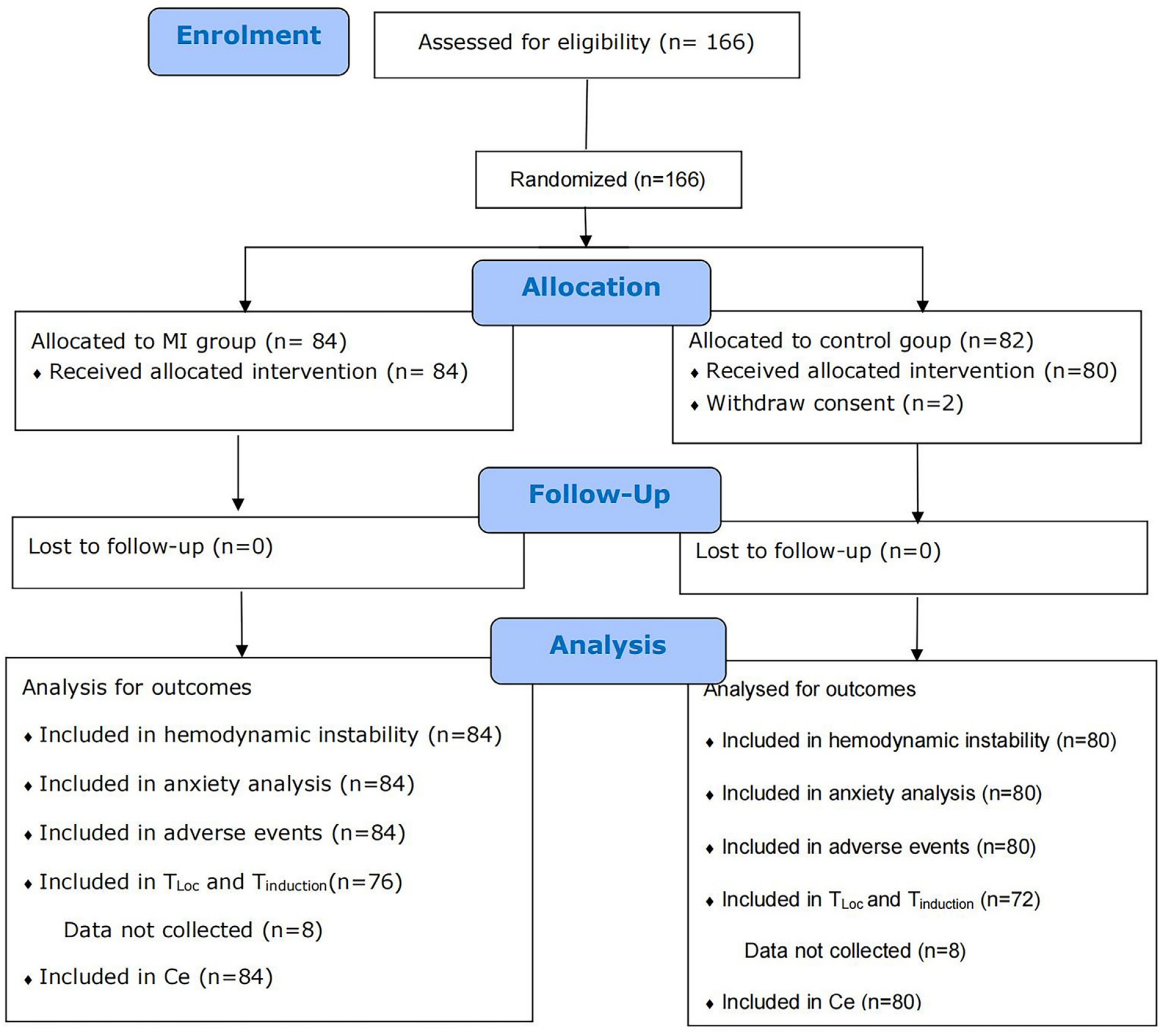
Patient recruitment flowchart.

Fig. 1 shows the patient recruitment flowchart. The study enrolled 166 patients and randomized to either the MI (*n* = 84) or the Control (*n* = 82). Two patients withdrew their consent in the control group. A total of 164 patients were included in the final analyses, with 84 in the MI group and 80 in the control group. Table 1 presents the characteristics of the patient cohort stratified by intervention. No significant difference was shown in age, American Society of Anesthesiologists rating, surgical history, chronic disease history, surgery type, or admission time. No patient felt discomfort, and no side effects were recorded in the waiting area.

**Table 1:** Baseline characteristics of recruited patients.

**Characteristics**	**Total** **(*n* = 164)**	**MI** **(*n* = 84)**	**Control** **(*n* = 80)**	**p-value**
Age, Mean (SD), y	44.1 (± 10.7)	43.3 (± 10.4)	44.8 (± 10.9)	0.34
Height, Mean (SD), cm	161.3 (± 4.7)	161.8 (± 4.8)	160.8 (± 4.6)	0.09
Weight, Mean (SD), kg	58.2 (± 7.4)	58.1 (± 6.9)	58.4 (± 7.9)	0.95
ASA, n (%)				
1	116 (70.7)	61 (72.6)	55 (68.8)	0.61
2	48 (29.3)	23 (27.4)	25(31.2)	
Operation History, n ( %)				
No	97 (59.1)	54 (64.3)	43 (53.8)	0.2
Yes	67 (40.9)	30 (35.7)	37(46.2)	
History of Chronic Disease^a^, n (%)				
No	129 (78.7)	67 (79.8)	62 (77.5)	0.86
Yes	35 (21.3)	17 (20.2)	18 (22.5)	
Operation Type, n (%)				
Thyroid	36 (22.0)	20 (23.8)	16 (20.0)	0.12
Breast	71 (43.3)	31 (36.9)	40 (50.0)	
Gynecology	53 (32.3)	29 (34.5)	24 (30.0)	
Others	4 (2.4)	4 (4.8)	0 (0.0)	
Admission Time, n (%)				
8:00‒12:00	101 (61.6)	47 (56.0)	54 (67.5)	0.12
12:00‒17:00	57 (34.8)	35 (41.7)	22 (27.5)	
17:00	6 (3.7)	2 (2.4)	4 (5.0)	

a History of chronic disease included well-controlled hypertension, diabetes or hypothyroidism.

### Primary outcome

**Fig. 2:**
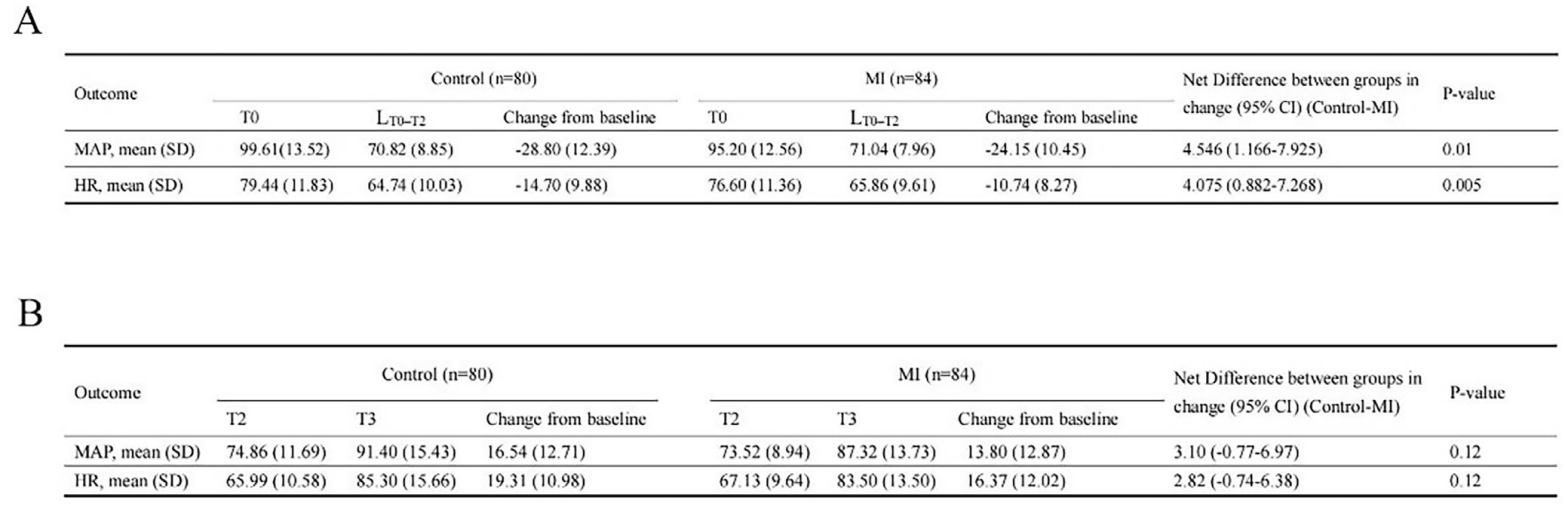
Incidence of hemodynamic instability during induction. (A) NCOVA analysis of the change in MAP and HR more than 20 % from T0 to LT0–T2 between the groups. (B) ANCOVA analysis of changes in MAP and HR from T2 to T3.

Table 2 summarizes all MAP and HR instabilities. The use of vasoactive drugs did not significantly differ between the groups (*p* = 0.09). ANCOVA revealed that the change in MAP by more than 20 % from T0 to LT0–T2 differed significantly between the groups (*p* = 0.01, Fig. 2A). The incidence of MAP instability during T0‒T2 was 69 % in the MI group and 83.8 % in the Control group (*p* = 0.03, Table 2). The 95 % CI of the rate difference (MI ‒ Control) for a change in MAP of more than 20 % from baseline demonstrated the potential superiority of the music intervention, as shown in Table 2 (95 % CI -0.2708 to -0.0164).

**Table 2:** Incidence of hemodynamic instability during anesthesia induction presented as n, percent, n (%).

**Events**	**MI, n (%)** **(n = 84)**	**Control, n (%)** **(n = 80)**	**Total, n (%)** **(n = 164)**	**Chi-Square**	**p-value**	**Rate difference (MI-Control)**	**95 % CI**
**Before intubation (T0‒T2)**							
MAP decrease > 20 %	58 (69.0)	67(83.8)	124 (75.6)	4.021	0.03	-0.147	[-0.2708, -0.0164]
HR decrease > 20 %	24 (28.6)	35 (43.8)	59 (36.0)	4.099	0.04	-0.1518	[-0.2904, -0.0049]
**During intubation (T2‒T3)**							
MAP increase > 20 %	36 (42.9)	39 (48.8)	75 (45.7)	0.5733	0.45		
HR increase > 20 %	43 (51.2)	58 (72.5)	101 (61.2)	7.865	0.005	-0.2131	[-0.3487, -0.0643]
**Vasoactive agents**							
Phenylephrine or ephedrine	15 (17.9)	23 (28.8)	38 (23.2)	2.731	0.09		
Atropine	2 (2.3)	0 (0)	2 (1.22)		0.50

**Fig. 3:**
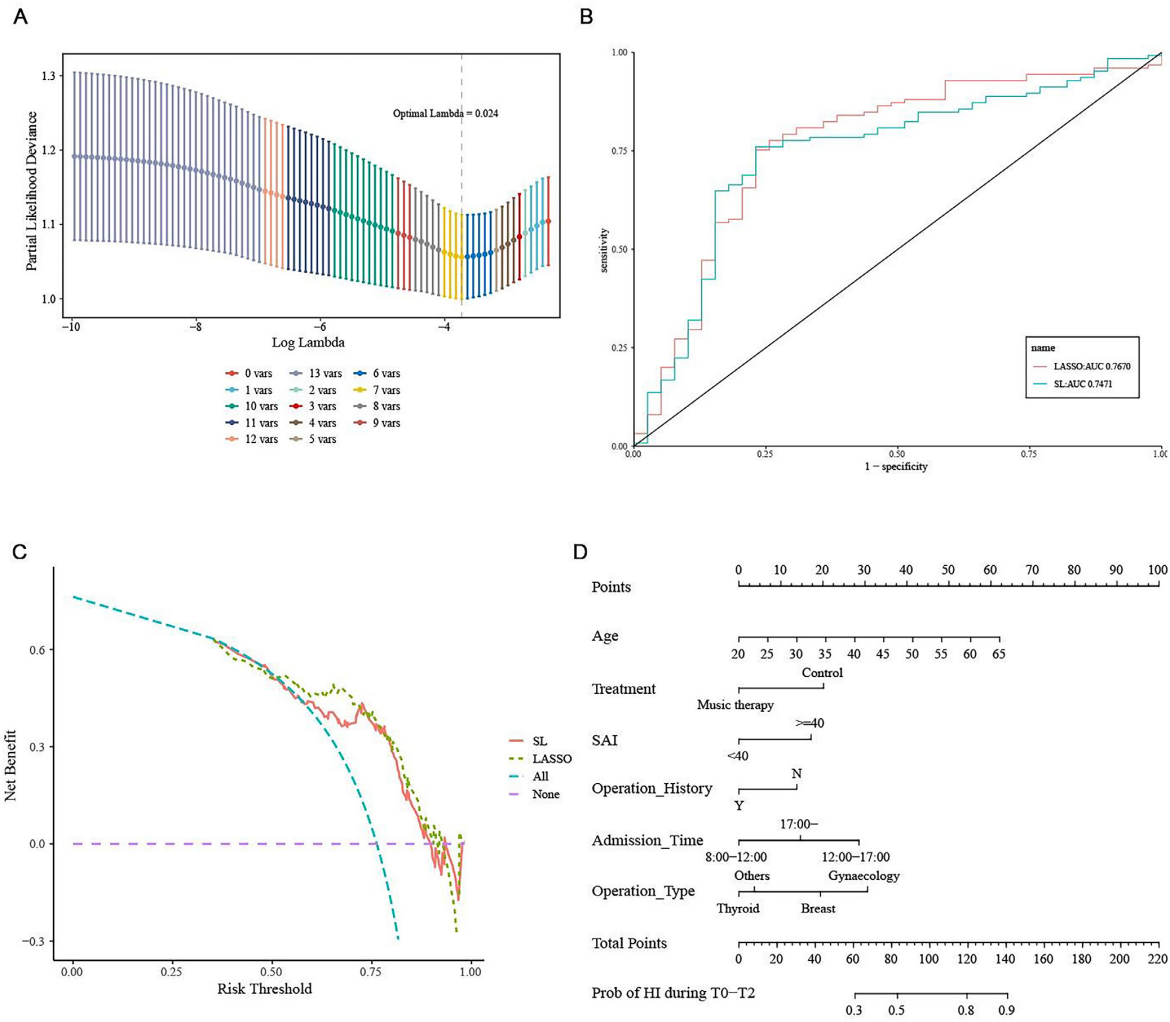
SL and LASSO regression models for the potential risk factor of MAP instability. **(**A) Relationship between partial-likelihood deviance and log(λ). The dotted line corresponds to the model of lambda.min (lambda = 0.024), which includes seven variables. (B) Receiver operating characteristic curves for two statistical methods for regression (Least Absolute Shrinkage and Selection Operator [LASSO] and Stepwise Logistic [SL]). (C) Decision curve analysis for the two models. The resulting curves are very close, indicating that the models are equal in prediction efficiency. (D) Nomogram of the logistic regression model predicting MAP instability.

Next, the authors constructed SL and LASSO regression models to investigate potential risk factors for MAP instability. Supplementary Figure S1 shows all variables in the model. The estimated coefficients were larger with SL regression than with LASSO regression. Those results suggest that music helped to stabilize hemodynamics during induction. Further, the SAI score of 40 or higher before induction was associated with a greater incidence of MAP instability (Fig. 3).

### Secondary outcomes

#### Incidence of HR instability during T0–T2

ANCOVA revealed that the change in HR more than 20 % from T0 to LT0–T2 differed significantly between the groups (*p* = 0.005, Fig. 2A). The incidence of HR instability greater than 20 % above baseline was lower in MI participants than in Control participants (*p* = 0.04, Table 2). The 95 % CI of the rate difference (MI ‒ Control) for HR change more than 20 % above baseline demonstrated the superiority of the music intervention (95 % CI -0.2904 to -0.0049).

#### HI during tracheal intubation (T2–T3)

ANCOVA revealed that changes in those values from T2 to T3 did not differ between the groups (HR: *p* = 0.12; MAP: *p* = 0.12, Fig. 2B). In this period, only two patients in the MI and one in the Control experienced a slightly decreased MAP (< 15 %). The authors therefore investigated the incidence of HI during intubation ‒ that is, an increase in MAP or HR of more than 20 % compared with values before intubation. MAP instability did not significantly differ between the groups, but HR instability was lower in MI participants (MAP: *p* = 0.45; HR: *p* = 0.005; Table 2).

#### Preoperative anxiety

**Fig. 4:**
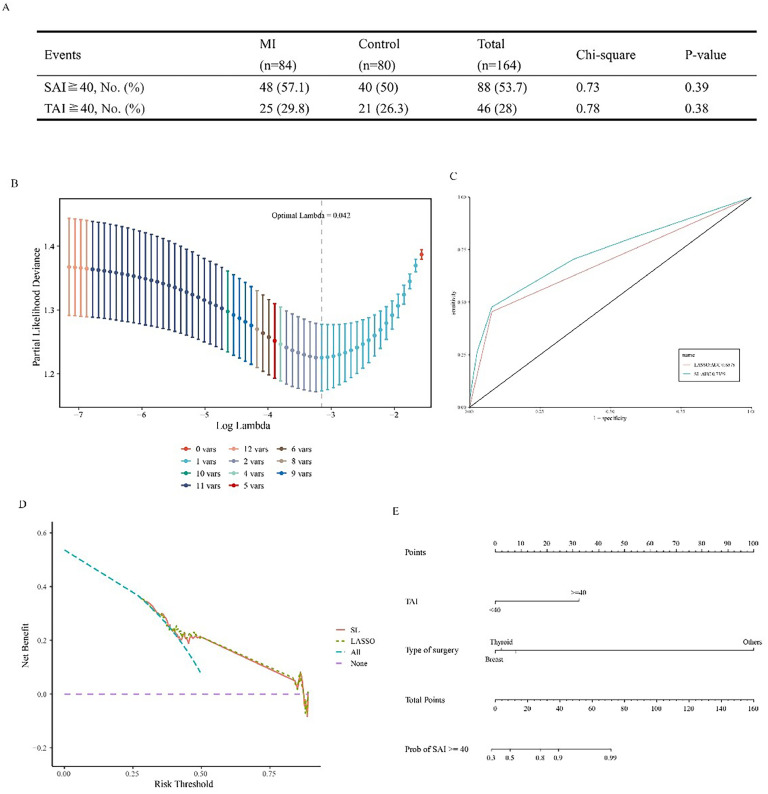
Preoperative anxiety. (A) Incidences of State Anxiety Inventory (SAI) and Trait Anxiety Inventory (TAI) scores of 40 or higher for patients in the waiting area. No significant difference between the groups was found by the chi-squared test. (B) Relationship between partial-likelihood deviance and log(λ). The dotted line corresponds to the model of lambda.min (lambda = 0.042), which includes two variables. (C) Receiver operating characteristic curves for two statistical methods of regression (Least Absolute Shrinkage and Selection Operator [LASSO] and Stepwise Logistic [SL]). (D) Decision curve analysis for the two models. The resulting curves are very close, indicating that the models are equal in prediction efficiency. (E) Nomogram of the logistic regression model predicting an SAI score of 40 or higher.

The incidences of SAI and TAI scores of 40 or higher at the first time point did not differ significantly between the groups (*p* = 0.39 and *p* = 0.38 respectively, Fig. 4A). In the waiting area, 88 participants scored 40 or higher on the SAI, and 46 scored 40 or higher on the TAI. The overall preoperative anxiety incidence was 53.7 % (88/164).

The authors then conducted SL and LASSO regression analyses to find out factors related to anxiety. Supplementary Fig. S2 shows the variables entered into the regression. The estimated coefficients that resulted were larger with SL regression than with LASSO regression. Nomogram demonstrated that patients with preoperative TAI scores of 40 and higher were more prone to have SAI scores of 40 and higher (Fig. 4B–E).

#### Effect of music on preoperative anxiety

Table 3 presents the SAI and TAI scores for both groups. At baseline, the mean SAI was 37.4 ± 10.6 in the Control and 40.7 ± 11.3 in the MI. After the music intervention, the mean SAI declined to 33.0 ± 10.3 in the MI, which was significantly lower than the mean SAI in the Control (38.1 ± 13.1), for a between-group difference of 8.01 (*p <* 0.001) by ANCOVA. Similarly, the mean TAI was significantly lower in the MI (32.8 ± 6.7) than in the Control (35.3 ± 8.2), for a between-group difference of 3.7 (p *<* 0.001).

**Table 3:** STAI scores in the waiting area presented as mean (SD).

**Outcome**	**MI (n = 84)**			**Control (n = 80)**			**Net Difference between groups in change (95 % CI) (Control‒MI)**	**p-value**
	**Baseline**	**Before induction**	**Change from baseline**	**Baseline**	**Before induction**	**Change from baseline**		
SAI, mean (SD)	40.7 (11.3)	33 (10.3)	-7.7 (7.4)	37.4 (10.6)	38.1 (13.1)	0.7 (6.7)	8.0 (5.9-10.2)	<0.001
TAI, mean (SD)	36.8 (7.3)	32.8 (6.7)	-3.9 (5.9)	35.2 (7.5)	35.3 (8.2)	0.1 (3.2)	3.7 (2.3-5.1)	<0.001

Linear regression analyses were conducted to assess the effect of music on anxiety in certain subgroups. Supplementary Fig. S3 shows all variables entered into the model and their estimated coefficients. No variable was associated with sensitivity to a music intervention (Supplementary Fig. S3A–C).

#### Other outcomes

Supplementary Table S1 shows the physiologic variables, including MAP, HR, and RR, measured in the preoperative waiting area for both groups. The baseline MAP was 97.9 mmHg in the Control and 93.7 mmHg in the MI. After the music intervention, MAP declined significantly in the MI, yielding a between-group difference of 3.9 (*p <* 0.001). ANCOVA revealed that HR and RR were both significantly lower in the MI than in the Control (HR: *p* < 0.001; RR: *p* < 0.001).

The authors investigated whether intubation-related adverse events differed between the groups during induction (supplementary Table S2). Cough reaction or body movement during intubation were less frequent in the MI (*p* = 0.02), and no myoclonus, laryngospasm, bronchospasm, or secretion was observed in either group. No difficulty with mask ventilation occurred, and all participants were successfully intubated on the first attempt using the video laryngoscope. The TLOC and Tinduction did not differ significantly between the groups, nor did C*e* at LOC (Supplementary Table S2).

## Discussion

Patients undergoing even minor surgical procedures can experience notable anxiety, owing to unfamiliar sights and sounds.^19^ The prevalence of anxiety has been observed to be higher among women than among men.^20^ A previous study indicated that 72 % of women experience high anxiety and 67 % experience insomnia before surgery.^21^ During cardiac stress testing, high levels of anxiety have been associated with elevated hemodynamic reactivity in women, and in women less than 65 years of age, high anxiety has been associated with increased inducible ischemia.^22^ In the present study, 53.7 % of the women participating had SAI scores considered to show current anxiety, while only 28 % had TAI scores suggesting high levels of general anxiety, indicating that even individuals with a low predisposition to anxiety can become apprehensive and anxious over an upcoming surgery.

Music is an engaging, multifaceted, therapeutic mind-body treatment approach used to address a variety of physiologic and neurologic symptoms.^23^ In a recent trial, postoperative pain and opioid use were observed to be reduced in the 24 h post-surgery when patients were exposed to music during general anesthesia.^24^ In the present study, the preoperative anxiety level in women was significantly reduced, as evidenced by lower STAI scores after those women had undergone more than 30 min of music intervention. HR and NIBP have been widely used as dependent variables in studies of anxiety-associated behavior.^25^ In this research, significant decreases in MAP, HR, and RR were observed after the music intervention in the surgical waiting area.

Needing a surgical procedure is a relatively common and significant stressor for patients, and distress is typically highest during induction of general anesthesia.^26^ The range of physiologic symptoms associated with anxiety and panic ‒ such as tachycardia, sweating, flushing, and shaking ‒ has been suggested to be mediated through SNS activation.^27^ Increased SNS activity no doubt contributes to blood pressure elevation and, consequently, possibly to the development of cardiovascular hypertrophy and cardiac arrhythmias.^28^ In healthy volunteers, anxiety has been associated with profound elevations in both blood pressure and plasma noradrenaline.^29^ Even with relatively mild mental stress, the nerves that stimulate the heart are markedly and preferentially activated and have the potential to trigger cardiac arrhythmias and myocardial ischemia in patients with coronary artery disease.^30^

The present study is the first to demonstrate that a preoperative music intervention can reduce the incidence of HI during anesthesia induction. The cardiovascular system is highly responsive to psychological and behavioral states. Stress and anxiety can occasionally interfere with the performance of surgeries, inducing HI, observed as elevated NIBP and HR.^31^ Binns-Turner et al. proved a music intervention throughout the perioperative period that reduced MAP, anxiety, and pain in women undergoing mastectomies for breast cancer.^32^ Kahloul and colleagues found more stable hemodynamic profiles in an MI receiving general anesthesia before an abdominal surgery.^33^ A systematic review indicated that listening to music might mitigate physiologic responses such as RR and systolic blood pressure in mechanically ventilated patients in critical care units.^34^ The present study found that the incidence of changes in MAP to more than 20 % above baseline was significantly decreased in MI participants compared with Control participants, as well as HR instability greater than 20 % above baseline during T0‒T2. Furthermore, the 95 % CI of the rate difference (MI−Control) demonstrated the superiority of music intervention in preventing hemodynamic fluctuations during anesthesia induction (95 % CI for MAP instability: -0.2708 to -0.0164; 95 % CI for HR instability: -0.2904 to -0.0049). However, during the intubation period, the authors didn't find the advantage of music intervention in blood pressure fluctuations, while the HR instability was still lower in MI participants.

Anxiety is typically associated with a predominance of SNS activity, leading to an increase in HR. Several studies have observed that listening to music significantly reduces HR.^35^ However Conrad et al. reported that music listening did not alter HR.^36^ The explanation for those contradictory reports might be that HR rises when music evokes higher levels of emotional arousal and that HR slows when music is more tranquilizing.^37^ One published study suggested that favorite and familiar music can relax patients and reduce muscle tension.^38^ The authors allowed patients in the MI to select their preferred music genres and observed that the incidence of HR instability was decreased in that group not only before intubation, but during intubation.

Despite evident clinical benefits, the operational mechanism of music therapy is not clearly understood. A functional magnetic resonance imaging study reported that stress activates the cingulate, prefrontal, and insular regions of the brain, correlating with exaggerated blood pressure reactivity.^39^ It seems that the influence of music on arterial blood pressure is related to molecular changes involving opiate and cytokine processes.^40^ Nilsson reported that listening to music encourages relaxation by increasing oxytocin levels.^38^

The present study has several limitations. First, the nature of the music intervention made it hard to implement blinding for the study participants. However, the patient self-rated STAI scores and the procedures for general anesthesia and data recording and analysis were entirely blinded, which might have helped to minimize the risk of bias. Second, the study cohort consisted of younger and well-educated patients. Older patients (> 65-years) attending these centers were rarely well educated, which makes understanding and completing the STAI difficult, thus influencing the analysis of anxiety levels. Given the limitations of the patient population, the present results might not be easily generalizable to other populations. Finally, the protocolized anesthesia technique might limit the generalizability of the hemodynamic findings.

## Conclusions

The present data suggest that a music intervention is a safe, effective, and inexpensive means of ameliorating hemodynamic fluctuations during anesthesia induction and that such an intervention significantly reduces preoperative anxiety for women who underwent elective non-cardiac surgery.

## Trial Registration

The study is registered in the Chinese Clinical Trial Registry (ChiCTR2000040254, http://www.chictr.org.cn/showprojen.aspx?proj=64383) (Trial period: Jan 1, 2021 ‒ Dec 31, 2022).

## Institutional review board statement

The study was conducted according to the guidelines of the Declaration of Helsinki, and approved by the Institutional Review Board of the Ethical Committee of Zhongshan Hospital, Fudan University on 13 November 2020 (Ethical Committee B2020-315(4)).

## Informed consent statement

Informed consent was obtained from all subjects involved in the study.

## Availability of data and materials

Participant data are stored in a ResMan Research Manager in accordance with the General Data Protection Regulations. Published data from this study cannot be traced to specific patients. The datasets generated and analyzed during the current study are not publicly available but are available from the corresponding author on reasonable request.

## Author's contributions

Conceptualization: Changhong Miao; Jing Zhong; Wanxia Xiong.

Data curation: Jie Wang; Wannan Chen; Linghui Jiang.

Formal analysis: Wanxia Xiong; Linghui Jiang

Funding acquisition: Wanxia Xiong; Changhong Miao; Jing Zhong; Zhiyao Wang

Investigation: Linghui Jiang

Methodology: Wanxia Xiong; Jing Zhong

Project administration: Changhong Miao; Wanxia Xiong; Jing Zhong.

Resources: Jie Wang

Software: Wanxia Xiong

Supervision: Linghui Jiang

Validation: Wannan Chen

Visualization: Wanxia Xiong

Writing – original draft: Wanxia Xiong

Writing – review & editing: All authors.

## Funding

This project was supported by the 10.13039/501100001809National Natural Science Foundation of China (n° 81801376, 81901130, 81873948, 81971868), and Shanghai Academic/Technology Research Leader Program (20XD 1423000). The funders had no role in the design of the study, collection, management, analysis and interpretation of the data; preparation, review, or approval of the manuscript and the decision to submit the manuscript for publication.

## Declaration of competing interest

The authors declare no conflicts of interest.

## Appendix Supplementary materials

Image, application 1
