# Supplementary material for: Effect of music on hemodynamic fluctuations in women during induction of general anesthesia: A prospective randomized controlled multicenter trial
Source: Clinics (Sao Paulo). 2024 Aug 2;79:100462. doi: 10.1016/j.clinsp.2024.100462 (PMC11345336; doi:10.1016/j.clinsp.2024.100462)

CLINICS-D-24-00329_Suplementary Material

**Supplementary Table S1** Physiological measures in the waiting area presented as mean (SD).

| **Outcome** | **MI (n = 84)** | | | **Control (n = 80)** | | | **Net Difference between groups in change (95% CI) (Control‒MI)** | **p-value** |
| --- | --- | --- | --- | --- | --- | --- | --- | --- |
|  | **Baseline** | **Before induction** | **Change from baseline** | **Baseline** | **Before induction** | **Change from baseline** |  |  |
| MAP, mean (SD), mmHg | 93.7 (13.2) | 87.7 (11.2) | -6.0 (7.6) | 97.9 (12.6) | 94.8 (12.0) | -3.1 (7.1) | 3.94 (1.89‒5.99) | <0.001 |
| HR, mean (SD) | 76.8 (11.1) | 73.2 (10.1) | -3.7 (6.8) | 78.4 (12.7) | 77.6 (11.2) | -0.7 (6.4) | 3.33 (1.51‒5.15) | <0.001 |
| RR, mean (SD) | 19.9 (3.4) | 16.0 (3.2) | -3.9 (3.9) | 20.4 (3.3) | 19.1 (3.6) | -1.5 (4.3) | 2.95 (1.97‒3.92) | <0.001 |

**Supplementary Table S2** Other secondary outcomes.

|  | **MI (n = 84)** | **Control (n = 80)** | **Total (n = 164)** | **Chi-Squared** | **p-value** |
| --- | --- | --- | --- | --- | --- |
| I**ntubation related adverse events, n (%)** |  |  |  |  |  |
| Y | 8 (9.5) | 18 (22.5) | 26 (15.9) | 5.172 | 0.02 |
| N | 76 (90.5) | 62 (77.5) | 138 (84.2) |  |  |
| **T_Loc_^a^, mean (SD), s** | 77.5 (67.8) | 85 (61.5) |  |  | 0.48 |
| **T_induction_^a^, mean (SD), s** | 364 (145.0) | 360 (110.5) |  |  | 0.80 |
| ***Ce* at LOC, mean (SD), ug/mL** | 3.43 (1.0) | 3.43 (1.1) |  |  | 0.96 |

^a^ Non-normally distributed variables presented as median (IQR), and performed by Mann-Whitney *U* test.

**Supplementary Figure S1** Stepwise Logistic (SL) and Least Absolute Shrinkage and Selection Operator (LASSO) regression analyses to estimate the relative risk of Mean Arterial Pressure (MAP) instability. (A) LASSO regression process: Each colored line represents the regression coefficient of a variable. The number below the x-axis is the penalty value. As λ increases, the regression coefficient of each variable decreases. (B) Coefficients for each variable from the two models.


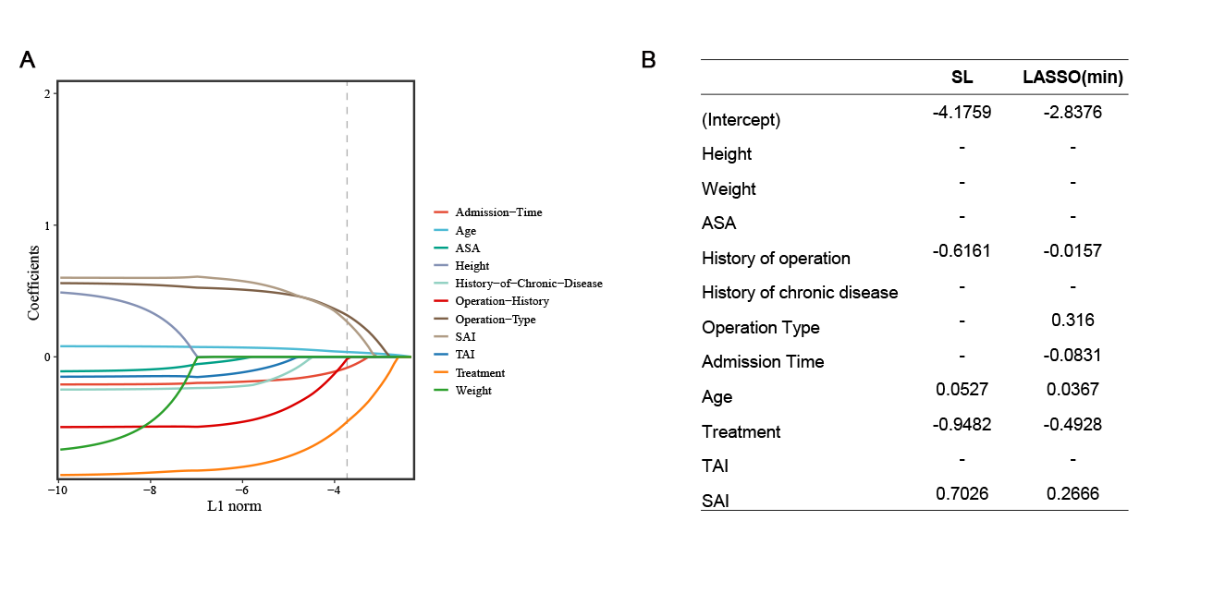


**Supplementary Figure S2** Stepwise Logistic (SL) and Least Absolute Shrinkage and Selection Operator (LASSO) regression analyses for preoperative anxiety. (A) LASSO regression process: Each colored line represents the regression coefficient of a variable. The number below the x-axis is the penalty value. As λ increases, the regression coefficient of each variable decreases. (B) Coefficients for each variable from the two models.


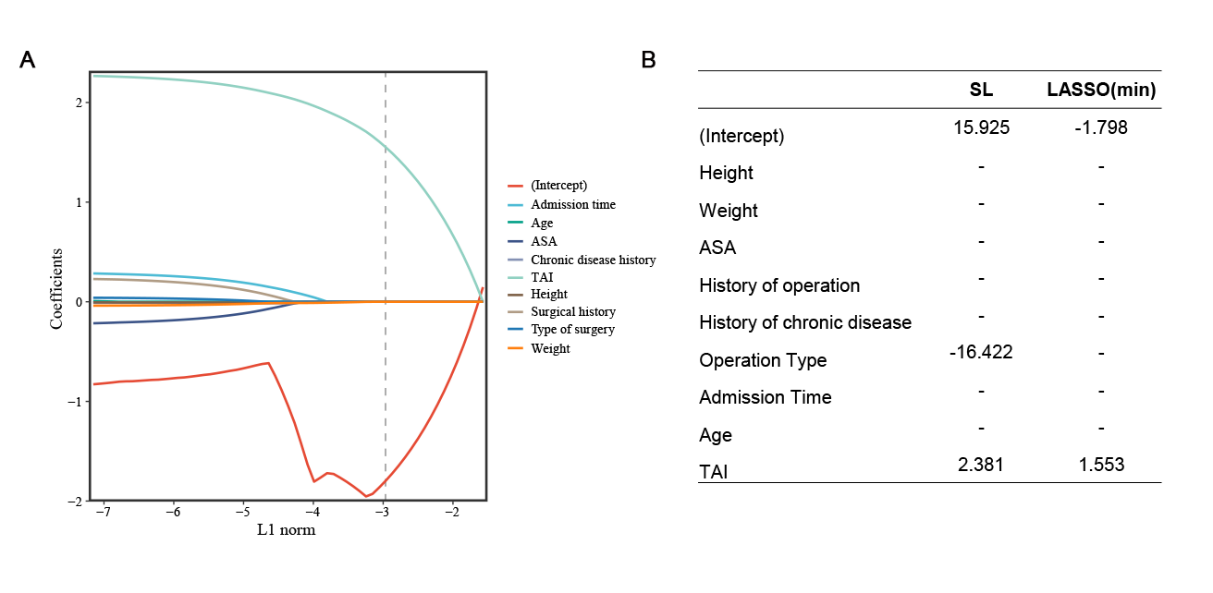


**Supplementary Figure S3** Linear regression analysis for changes in the State Anxiety Inventory (SAI) score in the MI group. (A) Regression coefficients for each variable. (B) Least Absolute Shrinkage and Selection Operator (LASSO) process: Each colored line represents the regression coefficient of a variable. The number below the x-axis is the penalty value. The λ increases as the regression coefficient of each variable decreases. (C) The relationship between partial-likelihood deviance and log(λ).


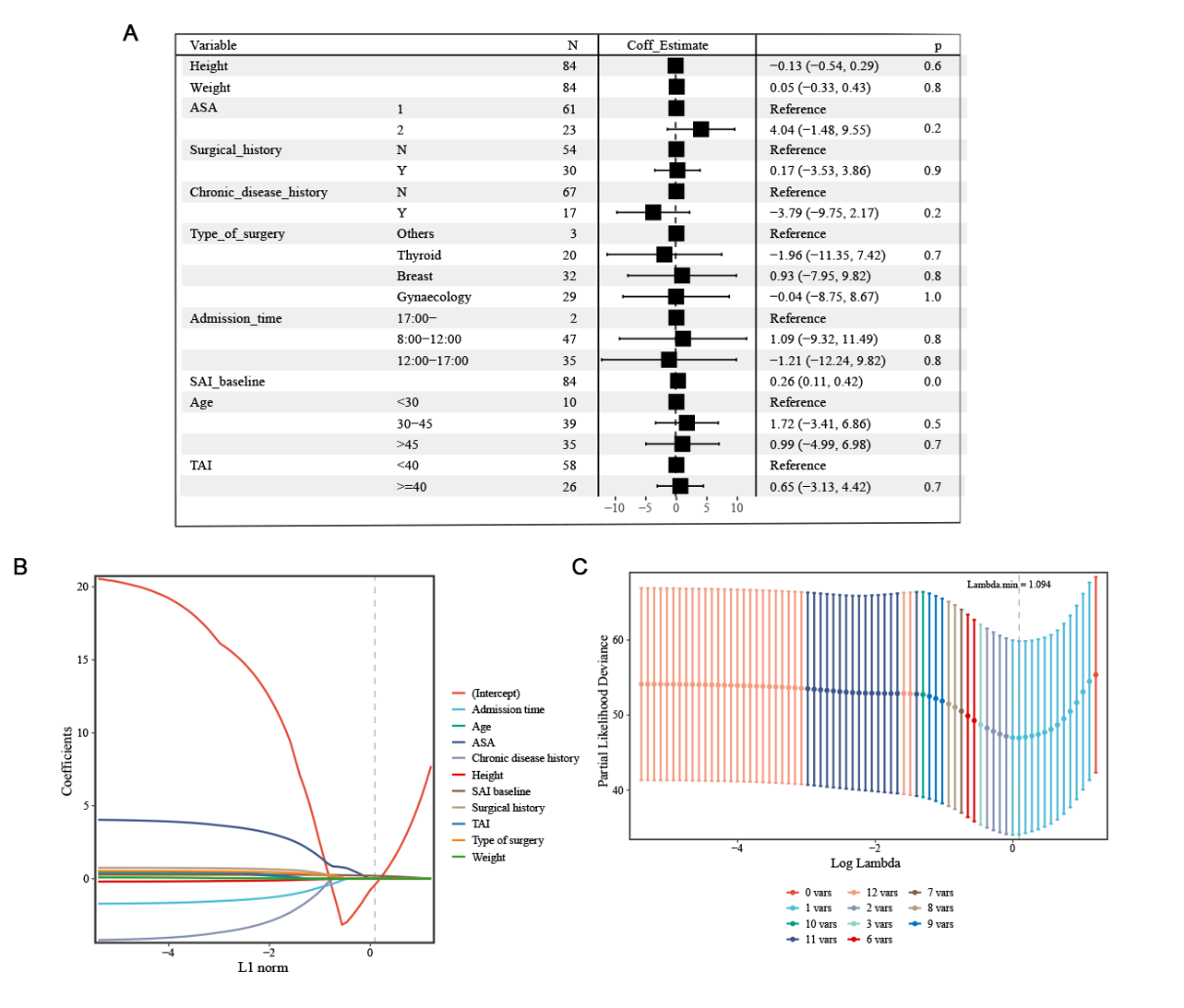

Supplement: Supplementary file 1 [file mmc1.docx]
